# Feasibility Study on a Portable Field Pest Classification System Design Based on DSP and 3G Wireless Communication Technology

**DOI:** 10.3390/s120303118

**Published:** 2012-03-06

**Authors:** Ruizhen Han, Yong He, Fei Liu

**Affiliations:** 1 College of Biosystems Engineering and Food Science, Zhejiang University, Hangzhou 310058, China; E-Mails: rzhan8403@163.com (R.H.); fliu@zju.edu.cn (F.L.); 2 College of Electronic Information, Zhejiang University of Media and Communications, Hangzhou 310018, China

**Keywords:** pest classification, image sensor, image processing, 3G network, DSP, Artificial Neural Network (ANN)

## Abstract

This paper presents a feasibility study on a real-time in field pest classification system design based on Blackfin DSP and 3G wireless communication technology. This prototype system is composed of remote on-line classification platform (ROCP), which uses a digital signal processor (DSP) as a core CPU, and a host control platform (HCP). The ROCP is in charge of acquiring the pest image, extracting image features and detecting the class of pest using an Artificial Neural Network (ANN) classifier. It sends the image data, which is encoded using JPEG 2000 in DSP, to the HCP through the 3G network at the same time for further identification. The image transmission and communication are accomplished using 3G technology. Our system transmits the data via a commercial base station. The system can work properly based on the effective coverage of base stations, no matter the distance from the ROCP to the HCP. In the HCP, the image data is decoded and the pest image displayed in real-time for further identification. Authentication and performance tests of the prototype system were conducted. The authentication test showed that the image data were transmitted correctly. Based on the performance test results on six classes of pests, the average accuracy is 82%. Considering the different live pests’ pose and different field lighting conditions, the result is satisfactory. The proposed technique is well suited for implementation in field pest classification on-line for precision agriculture.

## 1. Introduction

Pest control has always been considered the most difficult challenge to overcome in agriculture. Traditionally, pest management has been accomplished by means of a regular spray program which is based on a schedule rather than on the presence or likelihood of presence of insects in the field. More recently, growers have incorporated weather-based models to predict pest presence and apply control methods based on these models [1]. The most accurate method to control pests, and a method which is gaining interest in the wake of the need to minimize environmental impacts, is integrated pest management (IPM). The four main steps of IPM are detection, identification, application of the correct management and registration of the management [2]. The primary challenge with those steps is the identification. Classification of insect species can be extremely time consuming and requires technical expertise, so an automated insect identification method is needed. Due to the rapid development of digital image technology, there is a growing tendency in the field of agricultural research towards using machine vision technology to help the research and solve problems. In recent years, the use of artificial neural networks (ANN) has spread to many branches of science. Image analysis and ANN provide a realistic opportunity for the automation of routine species identification [3]. Do *et al.* [4] utilized various artificial neural networks to identify spider species using only digital images of female genitalia and achieved an average species accuracy of 81%. Artificial neural networks based on morphometric characters have been already applied in insect identification. Vanhara *et al.* [5] tested the methodology of ANN identification in the family Tachinidae on the basis of five model species of two genera, using 16 morphometric characters. Fedor *et al.* [6,7] identified Thysanoptera species using artificial neural networks with the morphometric characters. Russell *et al.* [8] developed an on-line automated identification system called SPIDA. The SPIDA system is trained to identify the 121 species of the Australasian spider family *Trochanteriidae* based on an artificial neural network model. SPIDA is currently available on the Internet, and users can submit their own images of spiders for classification, although some expertise and equipment is required to obtain optimal images. Murarikova *et al.* [9] confirmed the power of ANN by two independent non-numerical methods (molecular analysis, comparative morphology).

Most of the existing systems are semi-automated and all these systems have been trained on images taken from dead specimens. In a laboratory, dead specimens can be carefully positioned and photographed under consistent and ideal lighting conditions. In the field, however, live specimens may not adopt the ideal pose required, they may move when the image is being captured, and the lighting conditions outside the lab may be poor and may change unpredictably as a series of images are taken. This tends to make the classification task much more difficult.

Mayo and Watson [10] described different classifiers and datasets to identify live moths automatically and indicated that the best classifier is Support Vector Machine which achieved approximately 85% accuracy without manual preprocessing of the images. However, in those systems, the process of the training and testing was done in the laboratory and they can’t classify insects in real-time on-site. In order to detect the insects earlier, we aim to develop an on-line automated live insect identification system, which is portable and can provide the classification results in the field. The main research objectives of this paper were:

1. To design a hardware platform to implement image capturing, image processing, pest classifying with an Artificial Neural Network (ANN) classifier and image encoding.
2. To process the images and to conduct pest classification in DSP.
3. To design a wireless communication protocol and to transmit the images with a 3G network.
4. To display and store the pest images for expert precise classification and to design a host control platform for completing image decoding.
5. To test the designed system in the field.

This paper is organized as follows: Section 2 discusses the principles and algorithm flow of artificial neural network (ANN). Section 3 presents the hardware and software design of this system. Section 4 is devoted to the test of the proposed system. Field test results are provided in Section 5. Finally, conclusions are drawn in Section 6.

## 2. Artificial Neural Network

Artificial neural networks (ANNs) provide a way to emulate biological neurons to solve complex problems in the same manner to the human brain. For many years, especially since the middle of the last century, interest in studying the mechanism and structure of the brain has been increasing. In 1986, the Parallel Distributed Processing (PDP) research group published a series of algorithms and results and presented an ANN training algorithm named Back Propagation (BP) [11,12]. This BP training algorithm implemented with the general delta rule gave a strong impulse to subsequent research and resulted in the largest body of research and applications in ANNs although many other ANN architectures and training algorithms have been developed and applied simultaneously.

**Figure 1.**
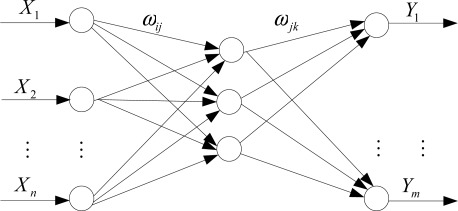
ANN network structure.

The massively parallel architecture of the ANN consists of multiple layers of simple computing elements with many interconnections between the layers. The computing elements are functionally analogous to neurons. They receive signals and in turn transmit a signal which is a function of the inputs. The function by which the inputs are evaluated may be a simple logic gate but more generally involves summation of weighted input signals. A transfer function is then applied to the weighted inputs to determine the output of the neuron. In this paper, we used a three-layer BP-ANN. Figure 1 shows the feedforward network between input *X* and output *Y*. In this paper, the BP-ANN was trained in advance via large numbers of experimental data. This training process was accomplished using Matlab language on a PC. After the BP-ANN was trained, the weights and thresholds were programmed in DSP for the BP-ANN model.

## 3. Prototype System Design and Implementation

### 3.1. Hardware Design

**Figure 2.**
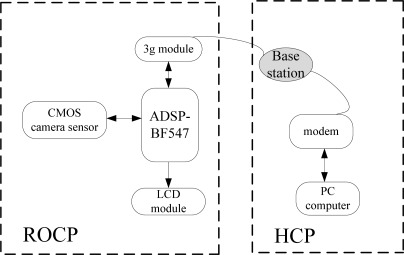
Architecture of the designed testing system.

The prototype system architecture adopted in this work is shown in Figure 2. This system includes a remote on-line classification platform (ROCP) and a host control platform (HCP). The ROCP mainly consists of a DSP, a 3G network module, an image sensor module, a LCD module and a power module. The HCP is composed of a PC and a modem for accessing the internet. The HCP can receive the image data send by the ROCP, decode them and display the image.

With the image sensor, we can get the pest images to the DSP in the ROCP platform. The DSP has two important things to do: on the one hand, it will preprocess the images, compute the features of the images, and give the initial classification results obtained from the BP-ANN classifier. On the other hand, it will encode the image data using JPEG 2000 and send them to the HCP through the 3G module. After receiving those image data, the HCP will then decode these data and display the pest images.

**Figure 3.**
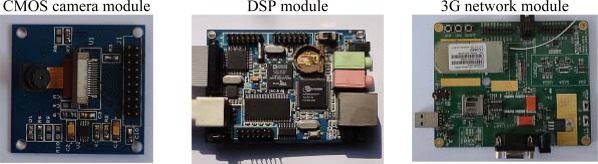
Photographs of the designed system including DSP module, CMOS camera module and 3G module.

This prototype system utilized an ADSP-BF547 processor as a kernel CPU in ROCP platform. The ADSP-BF547 processor is a member of the Blackfin family of products, incorporating the Analog Devices, Inc./Intel Micro Signal Architecture (MSA). The processor core clock is up to 600 MHz. It’s Dynamic Power Management provides the control functions to dynamically alter the processor core supply voltage to further reduce power consumption. Control of clocking to each of the peripherals also reduces power consumption. This is very suitable for portable appliances. The ADSP-BF547 processor peripherals include three SPI ports, eleven general-purpose timers with PWM capability, a real-time clock, a watchdog timer, a parallel peripheral interface, which is connected with the image sensor, an enhanced parallel peripheral interface which is connected with LCD module, and four UART ports, one of them is used to connect with the 3G module (module no: SIM5218A) for data transmission. The CMOS camera module (module no: OV9650) is used for pest image acquisition, the OV9650 is a color image sensor and has 1.3-Mpixel which is suitable considering the hardware resource and image resolution. Figure 3 shows photographs of the designed system.

### 3.2. Software Development

**Figure 4.**
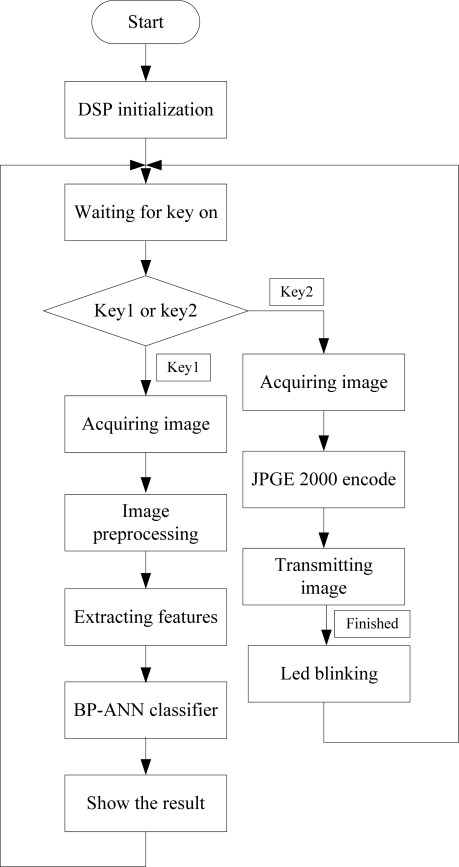
Flow diagram of the DSP program.

According to the hardware architecture of the designed portable system, the tasks of the whole system are the pest classification on DSP, image data compression coding, wireless data transmission, image decompression and image display on a PC. Therefore, software development of the system includes two parts—DSP software design and PC software design. The DSP programs are designed in three steps. Firstly, the data acquisition program acquires the image sensor response data. Secondly, DSP processes the image data, extracts the features and provides the classification results. Finally, it encodes the image data using JPEG 2000, packages them into different frames and sends them to a PC with the 3G module. The specific program flow diagram is shown in Figure 4. The image preprocessing is composed of image transforming, threshold processing, binarization and denoising. After finishing the image preprocessing, we extracted the image’s morphological characteristics including eccentricity ratio, sphericity and two Hu invariant moments for classification.

**Figure 5.**
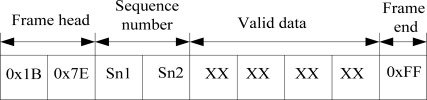
Wireless communication protocol.

**Figure 6.**
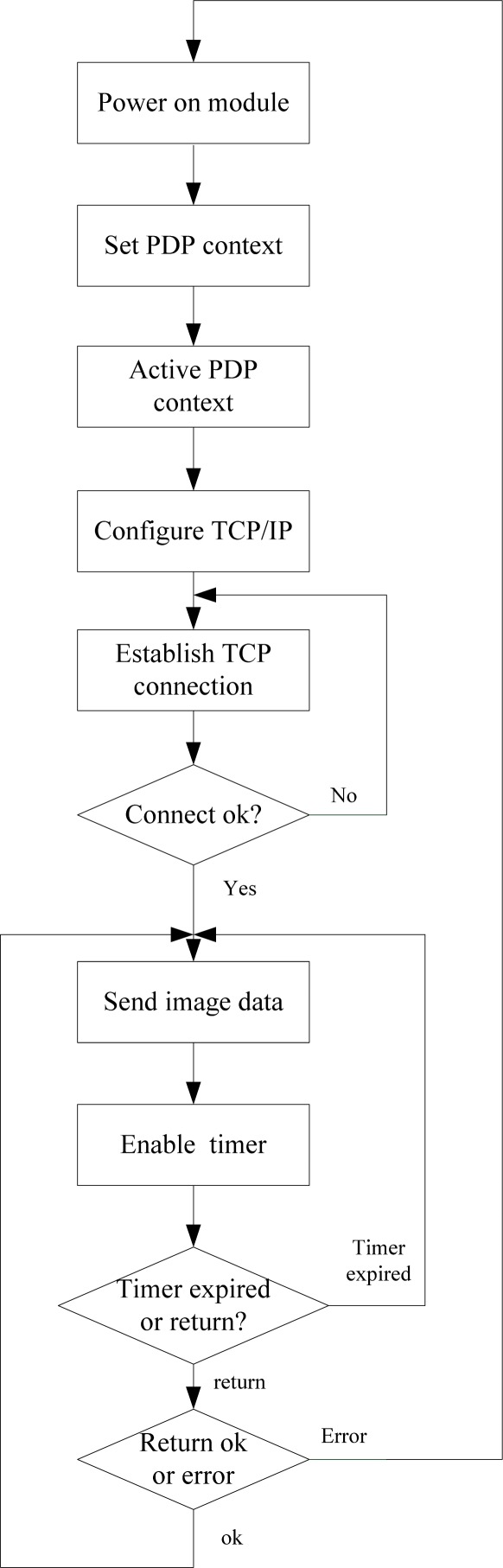
Wireless communication process.

In addition, we designed a wireless communication protocol and used the universal asynchronous receiver/transmitter (UART) interfaces of the DSP to carry out the serial data transmission between the 3G module and the DSP. The data frame format is composed of a frame head (0×1B, 0×7E), sequence number (two bytes), valid data bytes, and frame end (0×FF), as shown in Figure 5. Each frame has 512 valid data bytes. Communication baud rate is set at 57,600 baud. The flow diagram of the communication program of DSP is shown in Figure 6. After establishing the TCP/IP connection, we started to send the data and enable the timer which is used for avoid the system halting because of no return from the 3G module at the same time. If the timer expired and returned nothing, we resend the same data again. If the returned information is errorroneous, we reset the 3G module and establish the TCP/IP connection again. If we receive the right reply, we send next frame data until all data are sent.

The PC software of the HCP programmed in Visual C++ language decodes the image data, displays the images and stores the images.

## 4. Feasibility Study of the Designed Portable System

The feasibility study of the designed system was composed of three sections: DSP image acquisition and image processing tested the effect of image processing algorithm and extracted the morphology and color features. By training the BP-ANN, we obtained the weights and thresholds of the BP-ANN model. The data transmission authentication test validated the reliability of 3G network transmission.

### 4.1. DSP Image Acquisition and Image Processing Test

**Figure 7.**
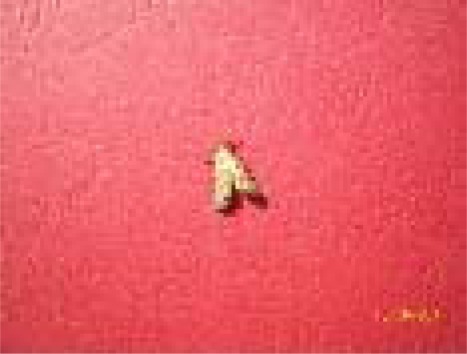
Image of *Cnaphalocrocis medinalis Guenee.*

The dataset used in this study is a library of live pest images created by the first author over a period of nearly a year. A pest trap was set up in the Fuyang Plant Protection Station (Zhejiang Province, China) and cleared every morning. Captured live pests were photographed and then released. *Cnaphalocrocis medinalis Guenee* is taken as an example and the image is shown in Figure 7.

**Figure 8.**
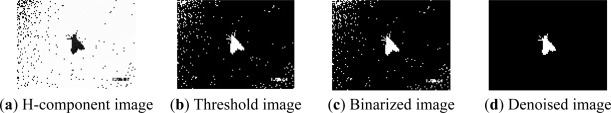
The image processing pipeline.

Considering that the trapped field pest’s morphological characteristics and color have relatively large differences, we extracted the morphology features and color features for classification. Geometrical features which describe the geometric properties of the target area are unrelated to the color value of the region. Therefore, the image is binarized before extracting it’s geometrical features. The Figure 8 depicts the automatic processing and feature extraction pipeline, using Figure 7 as an example input. The first step in feature extraction was to transform from the RGB color space to the HSV color space. Figure 8(a) depicts the results of the H-component when applied to the image of *Cnaphalocrocis medinalis Guenee* in Figure 7. The static threshold was obtained according to the statistics in the H-component, and was used for the input image and produced a threshold image as shown in Figure 8(b). Then the threshold image is binarized as shown in Figure 8(c). Finally, in order to reduce the noise, we adopted the method of searching the maximum linked area. We used the recursion method to find all connected region in which the value is “1”, and compared their size. The largest of them is the target object, the other is the noise. The result is shown in Figure 8(d). Now, a number of morphology features were calculated. The color features were described by color moments [13]. All features consisted of nine color moments, eccentricity ratio, sphericity and two Hu invariant moments which are invariant to image scaling, rotation and translation [14]. The color moments are defined by the following equations:

(1) $$E_{i}=\frac{1}{N}\sum_{j=1}^{N}p_{\mathrm{ij}}$$

(2) $$\sigma_{i}={\left(\frac{1}{N}\sum_{j=1}^{N}{\left(p_{\mathrm{ij}}-E_{i}\right)}^{2}\right)}^{\frac{1}{2}}$$

(3) $$s_{i}={\left(\frac{1}{N}\sum_{j=1}^{N}{\left(p_{\mathrm{ij}}-E_{i}\right)}^{3}\right)}^{\frac{1}{3}}$$

where *P_ij_* is the value of the *ith* color channel at the *jth* image pixel, *i* ∈ {1, 2, 3}, *N* is the number of image pixel.

The eccentricity ratio and the sphericity are defined by Equation (4) and Equation (5) respectively [15]. Two Hu invariant moments are defined by Equations (6) and (7) [14]:

(4) EC=p/q

where *p* and *q* are half the length of principal axis of momental ellipse:

(5) $$\mathrm{SP}=r_{i}/r_{c}$$

where *r_i_*, *r_c_* are the radius of the inscribed circle and the circumscribed circle of the target object respectively:

(6) $$\varphi_{1}=\eta_{20}+\eta_{02}$$

(7) $$\varphi_{2}={\left(\eta_{20}-\eta_{02}\right)}^{2}+4\eta_{11}^{2}$$

where *η_pq_* is the normalized central moments.

### 4.2. BP-ANN Model Training Process

The architecture of our BP-ANN was established according to the number of input neurons and the number of classifications. The initial ANN consisted of a layer of input neurons, a hidden-layer and a layer of output neurons, fully interconnected with the hidden-layer by random initial weights. Each input layer neuron corresponded to a feature. The number of nodes in the hidden-layer needs to be considered. As a preliminary selection, the optimum number of nodes in the hidden layer was determined by Equation (8) [16]:

(8) $$n_{1}=\sqrt{n+m}+a$$

where *n*_1_ is the number of nodes in the hidden layer, *n* is the number of input nodes, *m* is the number of output nodes, and *a* is an experiential integer from 1 to 10. By comparing the classification results of different models, we choose the model which has 13 input nodes, 10 nodes in the hidden-layer and 6 nodes in the output layer in the final model.

We selected six common field pests (*Cnaphalocrocis medinalis Guenee*, *Chilo suppressalis*, *Sesamia inferens*, *Naranga aenescens Moore*, *Anomala cupripes Hope*, *Prodenia litura*) for training the BP-ANN model. After acquiring and processing the images according to the procedure above from all samples, the set of images was divided into a training set and a test set. The composition of the training set is shown in Table 1. In order to remove the effects resulting from the difference of all features’ dimension, all features are normalized using the Equation (9) [17]:

(9) $$x_{\mathrm{ij}}^{,}=\frac{x_{\mathrm{ij}}-{\text{min}}_{j}}{{\text{max}}_{j}-{\text{min}}_{j}}\;\;\;\;\;\;\;\;(i=1,2,\cdots ,n;\;\;j=1,2,\cdots ,d)$$

where *n* is the number of pests and *d* is the number of features; *x_ij_* and *x′_ij_* are the non-transformed data and transformed data of the *jth* feature of the *ith* pest. max*_j_* and min*_j_* are the maximum and minimum of the *jth* feature in the all pests.

After the training was finished, the test was done according to the trained BP-ANN model. The composition of the testing set and the testing result are shown in Table 2.

### 4.3. Data Transmission Authentication Test

This test was conducted to verify the accuracy of the data sent and received during image data acquisition. The whole prototype testing system was implemented and placed at Zhejiang University Digital Agriculture and Agriculture Information Technology Research Center for conducting the test via China Unicom’s WCDMA network. Using the previously described test system and the transmission data format, the image data, after being processed by ADSP-BF547 which included in encoding, packaging, were sent to the HCP using the 3G wireless transmission module. Comparisons were performed to check transmission time and image data correctness. The received image data were stored on the PC hard disk. The actually image data were read from the SDRAM in ROCP. These data were then compared with the image data received by the HCP subsystem using the ultraedit software. The comparison shows that the received data are correct.

## 5. Results

After obtaining the weights and thresholds of BP-ANN, we programmed them in DSP for identification in the field. The CMOS image sensor OV9650 is used for image acquisition. The test was done in Fuyang plant protection station (Zhejiang, China). The number of test samples and the test results are shown in Table 3.

The performance of the trained BP-ANN in the testing runs demonstrated that the designed system was capable of identifying the common six pests, which were trapped at the Fuyang Plant Protection Station, with an overall average accuracy level of 82%. This level of accuracy was satisfactory given the complex field conditions and the limited amount of information on which the identification system was based. It should be emphasized that the set of analysed pests is rather a model example to demonstrate the potential of artificial intelligence in this area. In later work, we will study the identification of those pests which are not easily distinguishable by traditional taxonomic keys.

**Figure 9.**
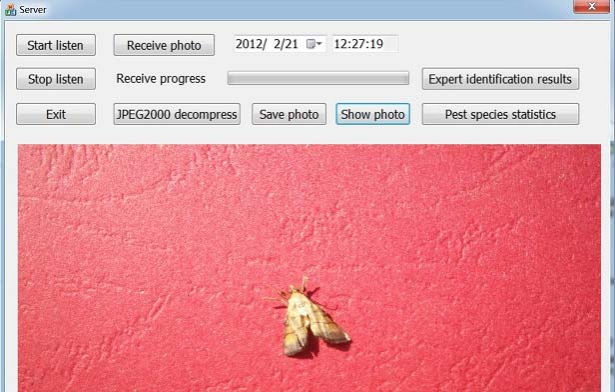
The GUI of HCP.

Figure 9 shows the GUI of the HCP; the photo, corresponding to the original image shown in Figure 7, is displayed by decompression. It is clear enough for experts to identify it.

## 6. Conclusions

Due to the need for pest identification in the field for precision agriculture, this paper studied the feasibility of on-line pest classification using machine vision technology. A DSP was used for this due to its powerful data processing functions. Considering the complex situation related to the field and the resource limitations of DSP, the classification achieved is satisfactory. Image data, which was encoded with JPEG 2000, was transmitted through the WCDMA network to an HCP for further identification. The test results show that the DSP can provide an initial result and the pest image in HCP is very clear and sufficient for further identification. The design of a reliable automatic pest classification system in HCP will be the focus our subsequent research efforts.

## Figures and Tables

**Table 1.** Composition of the training sets used to train BP-ANN.

**Species**	**Number**
*Cnaphalocrocis medinalis Guenee*	70
*Chilo suppressalis*	69
*Sesamia inferens*	72
*Naranga aenescens Moore*	70
*Anomala cupripes Hope*	75
*Prodenia litura*	76

**Table 2.** Testing results for BP-ANN.

**Species**	**Number**	**Accuracy (%)**
*Cnaphalocrocis medinalis Guenee*	23	83
*Chilo suppressalis*	15	80
*Sesamia inferens*	18	81
*Naranga aenescens Moore*	21	82
*Anomala cupripes Hope*	25	88
*Prodenia litura*	30	85
**Overall**	132	83

**Table 3.** Testing results for BP-ANN in the field.

**Species**	**Number**	**Accuracy (%)**
*Cnaphalocrocis medinalis Guenee*	20	82
*Chilo suppressalis*	18	79
*Sesamia inferens*	20	80
*Naranga aenescens Moore*	21	82
*Anomala cupripes Hope*	25	86
*Prodenia litura*	23	82
**Overall**	127	82
